# Correction: Associations of upper respiratory mucosa microbiota with Rheumatoid arthritis, autoantibodies, and disease activity

**DOI:** 10.1371/journal.pone.0310785

**Published:** 2024-09-16

**Authors:** Young Bin Joo, Junho Lee, Yune-Jung Park, So-Young Bang, Kwangwoo Kim, Hye-Soon Lee

The second author’s name is spelled incorrectly. The correct name is Junho Lee.
