# Supplementary figures and images for: Associations of upper respiratory mucosa microbiota with Rheumatoid arthritis, autoantibodies, and disease activity
Source: PLoS One. 2024 Aug 6;19(8):e0308010. doi: 10.1371/journal.pone.0308010 (PMC11302903; doi:10.1371/journal.pone.0308010)

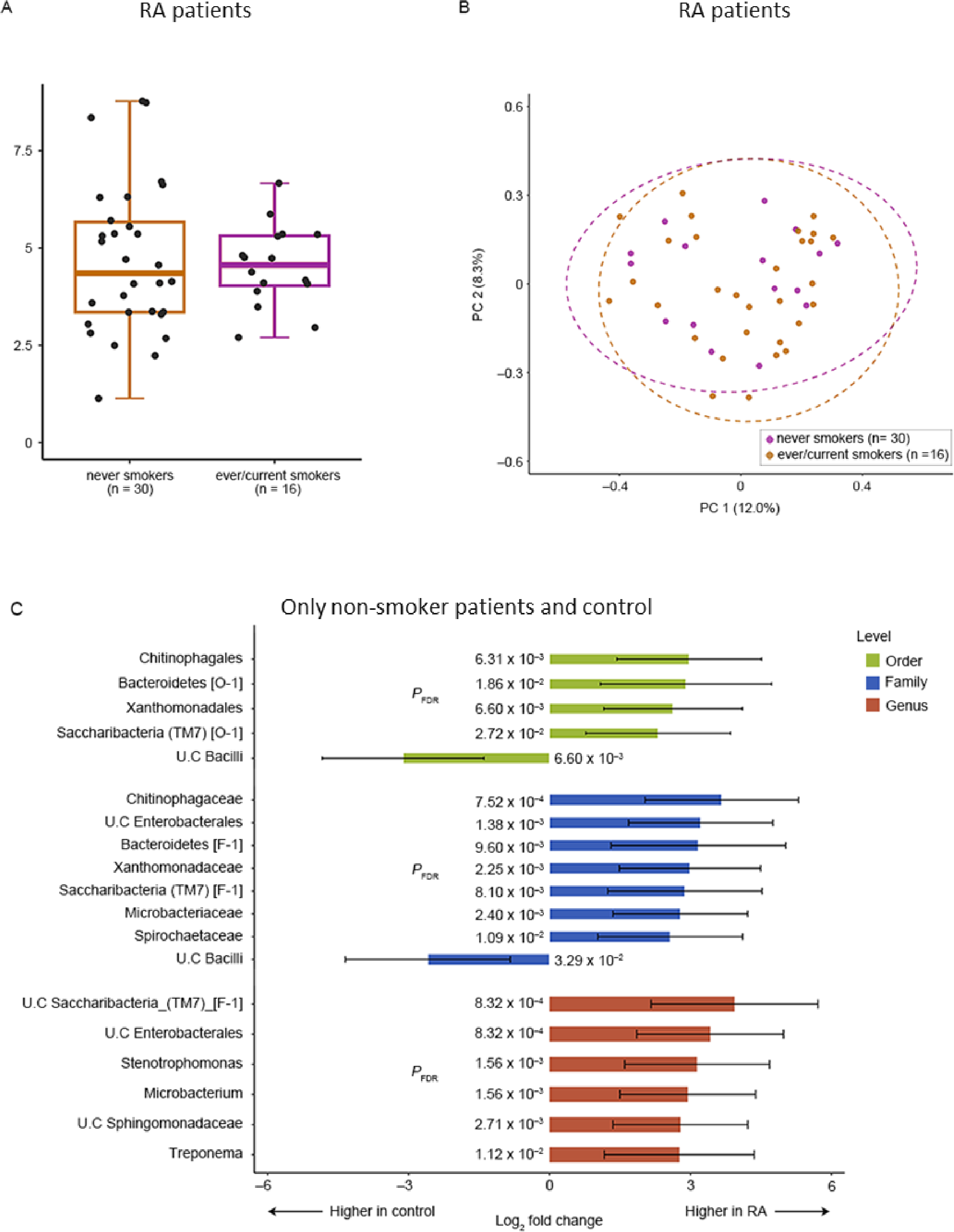

Supplement: S1 Fig — Alpha (A) and beta (B) diversity were compared between RA patients who had never smoked and those who had. Alpha diversity was evaluated using the Shannon index, while beta diversity was assessed using the Bray-Curtis dissimilarity. Differential abundance analyses of microbial composition were conducted between never smokers among RA patients and controls (C). Each bar in the figures represents the relative abundance of differentially abundant microbiota, along with the 95% confidence interval, and the colors indicate distinct taxonomic levels. The P value was adjusted for multiple testing using the Benjamini-Hochberg method. RA; rheumatoid arthritis, PC; Principal Coordinate, U.C; unclassified. (TIF) [file pone.0308010.s001.tif]

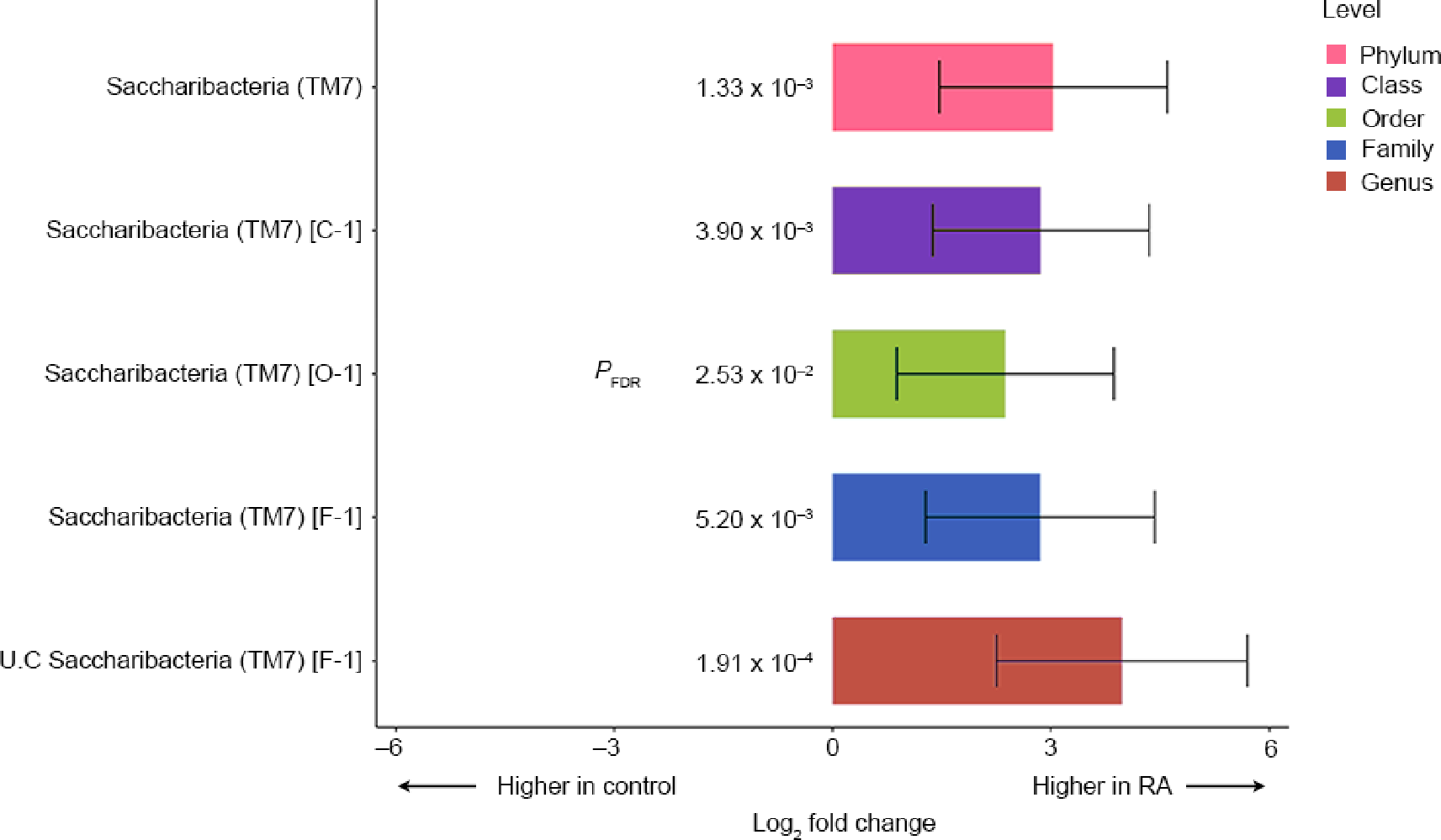

Supplement: S2 Fig — Each bar represents the relative abundance of differentially abundant Saccharibacteria, with 95% confidence intervals. Distinct taxonomic levels of Saccharibacteria are indicated by different colors. The P value was adjusted for multiple testing using the Benjamini-Hochberg method. RA; rheumatoid arthritis, U.C; unclassified. (TIF) [file pone.0308010.s002.tif]
