# Supplementary material for: Associations of upper respiratory mucosa microbiota with Rheumatoid arthritis, autoantibodies, and disease activity
Source: PLoS One. 2024 Aug 6;19(8):e0308010. doi: 10.1371/journal.pone.0308010 (PMC11302903; doi:10.1371/journal.pone.0308010)
Supplement: Attachment — Submitted filename: Response to Reviewers.docx [file pone.0308010.s003.docx]

**Responses to the reviewers' comments**

We are grateful to reviewer for their valuable comments, which we believe have helped us to greatly improve the manuscript entitled “Associations of upper respiratory mucosa microbiota with rheumatoid arthritis, autoantibodies, and disease activity” (PONE-D-24-15432).

Our manuscript has been thoroughly revised in accordance with the comments of the reviewer and the journal requirements. The sentences that have been changed in the text are written in red and underlined. We hope that the changes we have made adequately address the concerns of the reviewer, and that the manuscript will now be acceptable for publication.

--------------------------------Response to the comments of Journal requirements---------------------------

Journal Requirements:

When submitting your revision, we need you to address these additional requirements.

1. Please ensure that your manuscript meets PLOS ONE's style requirements, including those for file naming. The PLOS ONE style templates can be found at 
https://journals.plos.org/plosone/s/file?id=wjVg/PLOSOne_formatting_sample_main_body.pdf and 
<https://journals.plos.org/plosone/s/file?id=ba62/PLOSOne_formatting_sample_title_authors_affiliations.pdf>

**[Response]** We have revised our manuscript in accordance with the PLOS ONE guidelines for manuscript body formatting and title page requirements. These revisions include the reformatting of headings, figure captions, and the file naming for figures. Additionally, we have revised the references and the captions and file naming for supporting information to fully conform to the PLOS ONE journal format. We believe these changes meet the journal’s style requirements.

2. PLOS requires an ORCID iD for the corresponding author in Editorial Manager on papers submitted after December 6th, 2016. Please ensure that you have an ORCID iD and that it is validated in Editorial Manager. To do this, go to ‘Update my Information’ (in the upper left-hand corner of the main menu), and click on the Fetch/Validate link next to the ORCID field. This will take you to the ORCID site and allow you to create a new iD or authenticate a pre-existing iD in Editorial Manager. Please see the following video for instructions on linking an ORCID iD to your Editorial Manager account: https://www.youtube.com/watch?v=_xcclfuvtxQ

**[Response]** Thank you for your guidance regarding the ORCID iD requirement for the corresponding author. The corresponding author of the study have successfully authenticated a pre-existing ORCID iD within the Editorial Manager system.

3. Thank you for stating the following financial disclosure: "This research was supported by the Research Foundation of the Korean College of Rheumatology (YBJ), St. Vincent’s Hospital, the research institute of medical science (SVHR-2020-12) (YBJ), and the Basic Science Research Program through the National Research Foundation of Korea (NRF) funded by the Ministry of Education (NRF-2021R1A6A1A03038899) (HSL)." 
Please state what role the funders took in the study.  If the funders had no role, please state: ""The funders had no role in study design, data collection and analysis, decision to publish, or preparation of the manuscript."" 
If this statement is not correct you must amend it as needed. 
Please include this amended Role of Funder statement in your cover letter; we will change the online submission form on your behalf.

**[Response]** We have included a statement in the cover letter regarding the role of the funders, as recommended: “The funders had no role in study design, data collection and analysis, decision to publish, or preparation of the manuscript.”

4. We note that you have included the phrase “data not shown” in your manuscript. Unfortunately, this does not meet our data sharing requirements. PLOS does not permit references to inaccessible data. We require that authors provide all relevant data within the paper, Supporting Information files, or in an acceptable, public repository. Please add a citation to support this phrase or upload the data that corresponds with these findings to a stable repository (such as Figshare or Dryad) and provide and URLs, DOIs, or accession numbers that may be used to access these data. Or, if the data are not a core part of the research being presented in your study, we ask that you remove the phrase that refers to these data.

**[Response]** Thank you for your guidance regarding the data sharing requirements. In response to your comments, we have replaced the phrase “data not shown” with “S2 Fig” in the manuscript. We have prepared the corresponding supporting figure 2 and uploaded it via online submission.

5. Please amend either the abstract on the online submission form (via Edit Submission) or the abstract in the manuscript so that they are identical.

**[Response]** We have revised the abstract in the manuscript to match the version submitted online.

6. Please include captions for your Supporting Information files at the end of your manuscript, and update any in-text citations to match accordingly. Please see our Supporting Information guidelines for more information: http://journals.plos.org/plosone/s/supporting-information.

**[Response]** In accordance with the Supporting information guidelines, we have added captions for the supporting information at the end of the manuscript and included citations in the text.

----------------------------------Response to the comments of Reviewer #1------------------------------------

Responses to the comments of Reviewer #1

The study, titled 'Associations of upper respiratory mucosa microbiota with rheumatoid arthritis, autoantibodies, and disease activity,' aimed to investigate the association of upper respiratory microbiota with RA, autoantibody production, and disease activity. The study was conducted properly in general, and the writing is good. However, there are a few areas that need attention.

Major comments:

1) It’s not clear how the statistical analyses were conducted for the analyses of differential abundance, correlations, and baseline characteristics of the participants. The authors should provide a detailed description of the statistical analyses in the methods section.

**[Response]** Thank you for your comments on the clarity of the statistical analyses conducted in our study. We have provided a detailed description of the statistical methods used for the analyses of differential abundance, correlations, and the baseline characteristics of participants. This description has been included under the sub-heading “Statistical analysis” in the Method section as follows:

(Materials and Methods; on page 7)

**Statistical analysis**

To ensure the sensitivity of the diversity analysis, we used the rarefaction method to correct for different sequencing readout sizes of microbial data across samples before conducting diversity analysis. The alpha diversity of the upper respiratory microbiota was quantified using species richness, the Shannon index, and the Gini–Simpson index. Statistical differences in alpha diversity between the two groups were determined using the Mann–Whitney U test. The beta diversity, for examining differences in microbial composition among individual samples, was analyzed using principal coordinate analysis (PCoA) based on two distance metrics: the unique fraction (UniFrac) and Bray–Curtis dissimilarity. We assessed statistical differences in beta diversity between the sample groups using distance-based permutational multivariate analysis of variance (PERMANOVA) [14].

Differential abundance analysis of individual taxa between sample groups of interest (e.g., disease status, autoantibody, and disease activity markers) was performed using the DESeq2 software (version: 1.32.0) [15]. This analysis estimated log_2_-fold changes in normalized ASV counts, adjusting for both sex and age. The Benjamini-Hochberg procedure was applied to provide FDR-corrected p-values.

Pearson correlation coefficients were calculated to explore the potential association between the abundance of upper respiratory mucosa-associated taxa and various RA-related characteristics, such as disease activity markers and autoantibody titers among RA patients.

We compared the baseline characteristics of the RA patients and controls using an independent Student’s t-test for continuous variables and using Fisher's Exact test for categorical variables. All statistical analyses were performed using R (version 4.1.0).

2) Gene sequencing data used for this study are available at the NIH’s Sequence Read Archive (SRA) under accession number RJNA1058141. However, RJNA1058141 is not publicly available yet. The authors are required to make it publicly available before publication.

**[Response]** We have adjusted the release date for the amplicon sequencing data to May 31, 2024, and it is now publicly available.

Minor comments:

1) line 152, the citation format for ‘ref 14’ is wrong. The authors are advised to review the formatting of all references before publication.

**[Response]** We have revised “ref 14” to match the PLOS ONE formatting guidelines, specifically in accordance with the examples provided in the ICMJE guidelines (the samples of Formatted References for Authors of Journal Articles #44. Software on the Internet). Additionally, we reviewed the formatting of all references and revised them to comply with PLOS ONE style. Thank you for your comment.
